# A window of opportunity: Subdominant predators can use suboptimal prey

**DOI:** 10.1002/ece3.3139

**Published:** 2017-06-07

**Authors:** Kelly A. Jackson, Joshua S. McCord, Jennifer A. White

**Affiliations:** ^1^ Department of Entomology University of Kentucky Lexington KY USA

**Keywords:** Coccinellidae, cowpea aphid, exploitative competition, generalist predators, invasive species, niche partitioning

## Abstract

Introduced species have been linked to declines of native species through mechanisms including intraguild predation and exploitative competition. However, coexistence among species may be promoted by niche partitioning if native species can use resources that the invasive species cannot. Previous research has shown that some strains of the aphid *Aphis craccivora* are toxic to a competitively dominant invasive lady beetle, *Harmonia axyridis*. Our objective was to investigate whether these aphids might be an exploitable resource for other, subdominant, lady beetle species. We compared larval development rate, survival, and adult weight of five lady beetle species in no‐choice experiments with two different strains of *A. craccivora*, one of which is toxic to *H. axyridis* and one that is nontoxic. Two lady beetle species, *Cycloneda munda* and *Coleomegilla maculata,* were able to complete larval development when feeding on the aphid strain that is toxic to *H. axyridis*, experiencing only slight developmental delays relative to beetles feeding on the other aphid strain. One species, *Coccinella septempunctata,* also was able to complete larval development, but experienced a slight reduction in adult weight. The other two lady beetle species, *Hippodamia convergens* and *Anatis labiculata*, demonstrated generally low survivorship when consuming *A. craccivora*, regardless of aphid strain. All five species showed increased survival and/or development relative to *H. axyridis* on the “toxic” aphid strain. Our results suggest that this toxic trait may act as a narrow‐spectrum defense for the aphids, providing protection against only some lady beetle enemies. For other less‐susceptible lady beetles, these aphids have the potential to provide competitive release from the otherwise dominant *H. axyridis*.

## 1 INTRODUCTION

Competition often plays a large role in shaping community structure (Menge, 1976; Price & Kirkpatrick, 2009). Species that are the most successful in securing food and habitat resources can establish themselves as dominant species, potentially excluding other species that are less adept (Fretwell, 1969; Goldberg, 1987). For these subdominant species, survival in a community then becomes contingent on exploiting alternative resources that cannot or will not be utilized by the dominant species (Hill & Lodge, 1994; Messing & Wang, 2009). When a competitively dominant invasive species enters a novel ecosystem, it is able to disrupt established community interactions (Blossey & Notzold, 1995) and shift community composition (reviewed in Mooney & Cleland, 2001).

The multicolored Asian lady beetle, *Harmonia axyridis,* is a notably dominant invasive predator species (Roy et al., 2016). This beetle originated in Asia and has spread to at least 38 new countries since 1988 (Brown, Thomas et al., 2011). The ability of *H. axyridis* to rapidly expand its range and establish itself in novel communities is largely attributed to its success as a superior competitor and intraguild predator (Lucas, Gagne, & Coderre, 2002; Ware & Majerus, 2008). Additionally, high dispersal capability, multivoltinism, and the capability to survive in a wide variety of habitats have been hypothesized to contribute to the dominance of *H. axyridis* in novel ecosystems (Roy & Brown, 2015). Overall, native lady beetle populations and species diversity have been in decline (Harmon, Stephens, & Losey, 2007), and it is thought that *H. axyridis* has played a role in the decreasing biodiversity in some coccinellid communities (Bahlai, Colunga‐Garcia, Gage, & Landis, 2015; Brown, Frost et al., 2011).

*Harmonia axyridis* is an aphidophagous generalist, yet not all aphids are equivalently suitable food sources. For example, some aphids, such as *Megoura viciae* and *Aulacorthum magnolia*, cause delayed growth and mortality when consumed by the beetles (Fukunaga & Akimoto, 2007; Tsaganou, Hodgson, Athanassiou, Kavallieratos, & Tomanovic, 2004). Other aphids, such as the cowpea aphid, *Aphis craccivora*, vary in suitability as food for *H. axyridis* (Hukusima & Kamei, 1970; Kamo, Tokuoka, & Miyazaki,^2010^). Strains of *A. craccivora* originating from black locust, *Robinia pseudoacacia*, have been documented as toxic, inducing 100% mortality in *H. axyridis* larvae (Hukusima & Kamei, 1970; White, McCord, Jackson, Dehnel, & Lenhart, 2017). In contrast, *A. craccivora* strains that originated from alfalfa, *Medicago sativa*, are not toxic to *H. axyridis* larvae (White et al., 2017). This difference between strains is intrinsic to the aphids and is not a function of host plant chemistry. The mechanism of toxicity remains unknown, but aphid strains that originated from locust are consistently and heritably toxic to *H. axyridis*, even after many generations of rearing on alternate host plant species (White et al., 2017).

Despite the strong negative effects that locust‐origin *A. craccivora* have on *H. axyridis*, the toxicity might not be ubiquitous across coccinellid predator species. Previous studies on the suitability of *A. craccivora* as a food source have been conducted with other coccinellid species, often with results suggesting that they are acceptable prey that supports coccinellid development to adulthood (Ferrer, Dixon, & Hemptinne, 2008; Omkar & Mishra, 2005; Omkar & Srivastava, 2003). However, it is not clear which strains of the aphid were evaluated in these trials. These previous studies may have assayed *A. craccivora* strains on which *H. axyridis* would have performed well*,* or they may have assayed strains that would have been toxic to *H. axyridis*, which would indicate that these other lady beetles are less susceptible to toxic *A. craccivora*. In other words, the toxic trait may be broad spectrum against a wide range of coccinellid predator species, or narrow spectrum against only a subset of the predators.

Here, we examined whether locust‐origin *A. craccivora* is suitable food for several other coccinellid species. Understanding the specificity of toxicity in these aphids has important ramifications, both for predicting the defensive virtue of the trait for the aphid and community outcomes among coccinellids. If other coccinellid species can use these aphids as a food source, the presence of selectively toxic aphids in an environment could mitigate competitive differentials between *H. axyridis* and subdominant lady beetle species, facilitating niche partitioning, predator coexistence, and diversity.

## 2 MATERIALS AND METHODS

We evaluated the development and survival of five lady beetle species: *Anatis labiculata*,* Coccinella septempunctata*,* Coleomegilla maculata, Cycloneda munda*, and *Hippodamia convergens*. All species are native to N. America except *C. septempunctata*, which is native to the Palearctic. All species are also multivoltine habitat generalists commonly found in field crops, except *An. labiculata*, which is a univoltine arboreal species. Each species co‐occurs with both *A. craccivora* and *H. axyridis* in the field. Wild caught beetles were collected from Lexington, KY, USA in 2014 and 2015. The beetles were grouped by species and life stage in Petri dishes (100 × 25 mm) and maintained in an incubator at 25°C, 16‐hr:8‐hr light:dark, 65% humidity. Both juvenile and adult beetles were fed pea aphids (*Acyrthosiphum pisum*). When mating occurred, the paired male and female were removed from the colony and placed in their own Petri dish with folded paper for egg deposition. Egg papers were regularly removed from the parents’ Petri dish to prevent cannibalism.

All aphids (*A. craccivora* and *Ac. pisum*) originated from clones originally collected in Lexington, Kentucky, USA and were maintained in colonies on fava bean (*Vicia faba*) in the laboratory at ambient room temperature. *Aphis craccivora* clones were initially collected from either black locust or alfalfa as described in Wagner et al. (2015). To date, all clones collected from black locust are intrinsically toxic to *H. axyridis*, and all clones collected from alfalfa are nontoxic to *H. axyridis* (White et al., 2017). The toxicity status of the two aphid strains is unknown for non‐*Harmonia* coccinellid species; as such, hereafter locust‐origin *A. craccivora* will be referred to as L‐strain and alfalfa‐origin *A. craccivora* will be referred to as A‐strain.

For each lady beetle species, we compared beetle development time and survival on the two strains of *A. craccivora* in no‐choice experiments. Neonate larvae were removed from their egg mass before sibling cannibalism could occur and were placed individually in Petri dishes (35 × 10 mm) that had an excised circle of fava bean leaf embedded in 1% agar. We randomly assigned each larva to an aphid treatment. Three lady beetle species (*C. septempunctata*,* Co. maculata*, and *Cy. munda*) had one of two treatments: either L‐strain or A‐strain *A. craccivora*. Because the remaining two beetle species, *Hi. convergens* and *An. labiculata*, showed poor survival overall on *A. craccivora*, we included a third treatment of *Ac. pisum* aphids as a control. We fed the larvae their assigned aphid diets (mixed instars) *ad libitum* for the duration of development and monitored daily for mortality and developmental stage. Once the beetles reached the third instar, we moved them to larger Petri dishes (60 × 15 mm) and provided them with a cotton ball soaked in DI water along with their aphid treatment. For the beetles that survived to adulthood, teneral adults were allowed to sclerotize for 1 day before weighing to the nearest milligram. Sex of each adult beetle was determined through mating observations of the adults. Sex did not statistically affect the differences between treatments and was removed from subsequent analysis. Sample size varied among species based on availability of neonates for each species.

We compared survival to adulthood among treatments using Kaplan–Meier survival analysis followed by the Mantel‐Cox test. For species in which all individuals survived to adulthood in one or more treatments, Kaplan–Meier statistics could not be calculated and we instead used Fisher's exact test to compare survival between treatments. We similarly used Kaplan–Meier analysis to compare development time (time to pupation) among treatments, coding individuals that died as censored values. For *An. labiculata*, all individuals died in the L‐strain treatment, so we were only able to compare development time between the A‐strain treatment and the *Ac. pisum* control treatment. Finally, we compared adult weight between treatments using two‐sample *t*‐tests for each species. All weight data conformed to homoscedacity and normality assumptions. For *Hi. convergens*, only three adults were produced across the L‐ and A‐strain *A. craccivora* treatments, so we combined these two diet treatments for comparison with the *Ac. pisum* control treatment. For *An. labiculata,* we again only compared the A‐strain treatment to the *Ac. pisum* control treatment, as there were no adults produced in the L‐strain treatment. We conducted all statistical analyses in IBM SPSS v.24.

## 3 RESULTS

**Figure 1:**
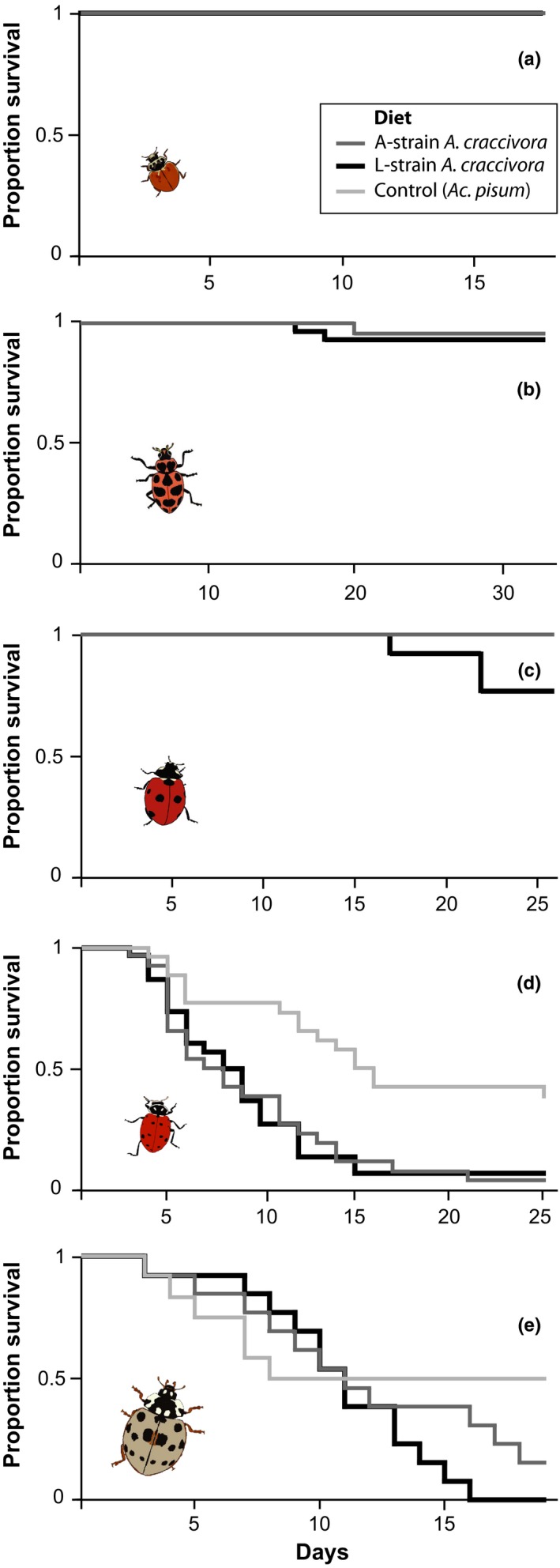
Survivorship of larvae of (a) *Cycloneda munda*, (b) *Coleomegilla maculata*, (c) *Coccinella septempunctata*, (d) *Hippodamia convergens*, and (e) *Anatis labiculata* lady beetles when fed on different aphids. All five beetle species included L‐strain and A‐strain *A. craccivora* treatments; L‐strain aphids cause rapid mortality of *Harmonia axyridis* larvae, A‐strain aphids do not. *Hippodamia convergens* (d) and *An. labiculata* (e) additionally included a control treatment of *Acyrthosiphum pisum* aphids. Trials were concluded when all beetles had reached adulthood or died

**Figure 2:**
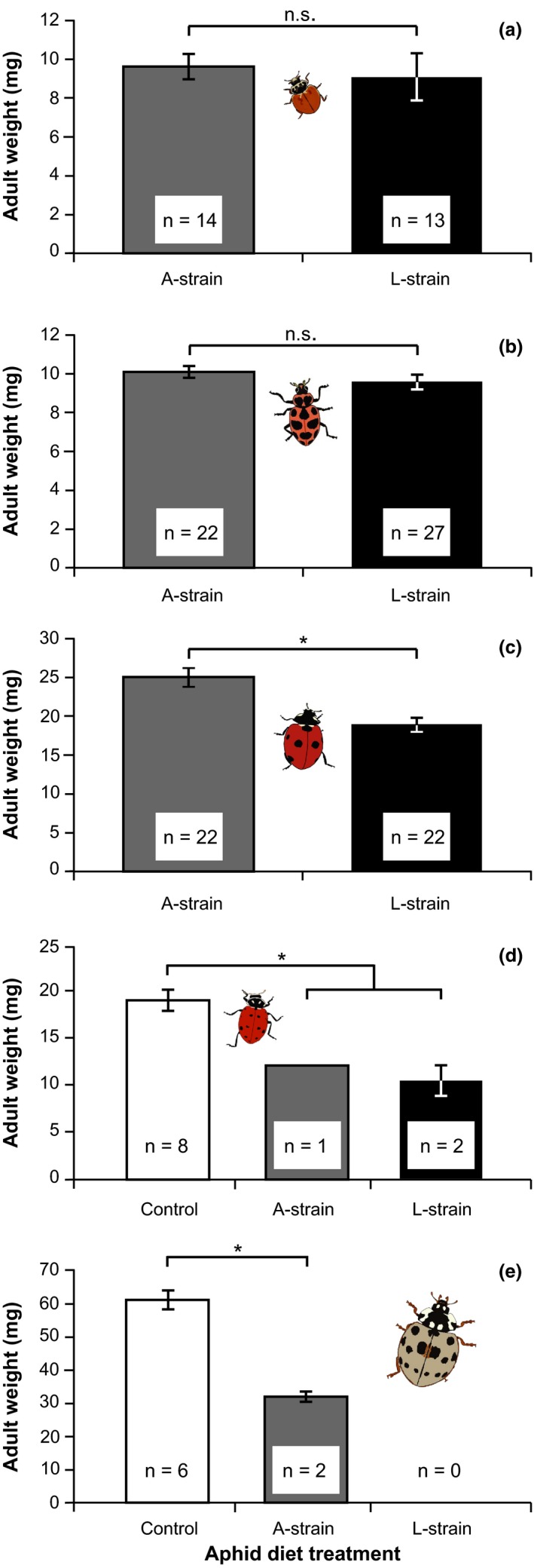
Mean ± 1 *SE* adult weight of (a) *Cycloneda munda*, (b) *Coleomegilla maculata*, (c) *Coccinella septempunctata*, (d) *Hippodamia convergens*, and (e) *Anatis labiculata* lady beetles when fed on different aphids. All five beetle species included L‐strain and A‐strain *A. craccivora* treatments; *Hi. convergens* (d), and *An. labiculata* (e) additionally included a control treatment of *Acyrthosiphum pisum* aphids. Sample sizes of beetles surviving to adulthood per treatment are inset within each column. Brackets indicate statistical contrasts: An asterisk indicates a contrast that was significant at *p* < .05, n.s. indicates a contrast that was not significantly different

In contrast to *H. axyridis*, which experiences 100% mortality when exposed to L‐strain *A. craccivora*, the tested lady beetle species showed a wide range of tolerance. Two species*, Cy. munda* and *Co. maculata*, were only slightly affected by the L‐strain of *A. craccivora*. For *Cy. munda*, all of the beetle larvae survived to adulthood when reared on either L‐strain (*n* = 13/13) or A‐strain (*n* = 14/14) aphids (Figure 1a; Fisher's exact test *p* = 1.0). *Cycloneda munda* larvae exhibited a slight delay in development when reared on L‐strain aphids, taking approximately 10% longer to pupate than those on A‐strain aphids (L‐strain mean ± *SE* = 11.7 ± 0.3 days, A‐strain = 10.7 ± 0.2 days; Mantel‐Cox χ^2^ = 8.4, *df* = 1, *p* = .008). However, there was no significant difference between the adult weights of *Cy. munda* reared on the two treatments (Figure 2a; *t* = .60, *df* = 25, *p* = .55). Similarly, *Co. maculata* experienced high survival on both L‐strain (*n* = 26/28 survived) and A‐strain (*n* = 23/24 survived) *A. craccivora* (Figure 1b; Mantel‐Cox χ^2^ = 0.04, *df* = 1, *p* = .84) and had 12% slower development on L‐strain than A‐strain *A. craccivora* (L‐strain = 18.5 ± 0.4 days, A‐strain = 16.8 ± 0.5 days; Mantel‐Cox χ^2^ = 4.24, *df* = 1, *p* = .049). There was no difference in adult weight of *Co. maculata* between treatments (Figure 2b; *t* = .84, *df* = 47, *p* = .40).

*Coccinella septempunctata* showed a moderately negative response to L‐strain *A. craccivora*. There was a trend toward reduced survival on L‐strain aphids, with 76.9% (n = 10/13) surviving, relative to 100% (n = 14/14) survival on A‐strain aphids (Figure 1c; Fisher's exact test *p* = .098). The beetles feeding on L‐strain aphids took approximately 2 days longer (15.6%) to pupate than beetles feeding on A‐strain aphids (L‐strain = 17.0 ± 0.8 days, A‐strain = 14.7 ± 0.2 days; Mantel‐Cox χ^2^ = 10.9, *df* = 1, *p* = .001) and showed a 24% reduction in adult weight (Figure 2c; *t* = 4.0, *df* = 22, *p* < .001).

The remaining two species, *Hi. convergens* and *An. labiculata*, had generally poor survival, and additionally included an *Ac. pisum* control treatment. *Hippodamia convergens* performed poorly in all treatments. On L‐strain aphids, only 6.7% (n = 2/30) survived, on A‐strain aphids only 3.8% (n = 1/26) survived, and on the control *Ac. pisum* aphids, 34.6% (*n* = 9/26) survived. Survival time was significantly longer on control aphids than either *A. craccivora* strain (Figure 1d; Mantel‐Cox χ^2^ = 15.6, *df* = 2, *p* < .001). Time to pupation also tended to be slower on either *A. craccivora* strain than the *Ac. pisum* control (L‐strain = 19 ± 1 days, A‐strain = 20.5 ± 0.4 days, control = 17.3 ± 0.9 days), but low survival numbers precluded statistical significance (Mantel‐Cox χ^2^ = 5.26, *df* = 2, *p* = .07). For these few survivors on *A. craccivora*, adult weight was 40% lower than on the *Ac. pisum* control (Figure 2d; *t* = 3.92, *df* = 9, *p* = .003), but could not be compared statistically between L‐strain and A‐strain aphid diets.

For *An. labiculata*, 0% (*n* = 0/13) of larvae survived to adulthood on L‐strain aphids, but only 15% (*n* = 2/13) survived on A‐strain aphids, and 50% (*n* = 6/12) survived on the *Ac. pisum* control. There was no difference in survival between beetles fed L‐strain versus A‐strain *A. craccivora* (Fisher's exact test *p* = .48), but the survival on any *A. craccivora* diet was significantly lower than the *Ac. pisum* control (Fisher's exact test *p* = .007). Time to death did not differ significantly among any of the treatments, due to some early mortality of beetles on control aphids, and relatively long larval survival on both *A. craccivora* strains before dying (Figure 1e; Mantel‐Cox χ^2^ = 3.75, *df* = 2, *p* = .15). On L‐strain aphids, *An. labiculata* larvae exhibited some development before dying: 54% of beetles survived into the second instar and 23% survived to the third. For the two beetles that survived to adulthood on A‐strain *A. craccivora*, time to pupation was 15% longer than the survivors on *Ac. pisum* (A‐strain = 17.4 ± 0.3 days, control = 15.1 ± 0.5 days; Mantel‐Cox χ^2^ = 8.10, *df* = 1, *p* = .004), and adult weight was nearly 50% lower (Figure 2e; *t* = 5.43, *df* = 6, *p* = .002).

## 4 DISCUSSION

Coccinellid species varied in their ability to use L‐strain *A. craccivora*. Three species, *Cy. munda, Co. maculata,* and *C. septempunctata,* showed only slight negative effects of consuming L‐strain versus A‐strain *A. craccivora*: All or most beetles survived to adulthood feeding on L‐strain aphids and exhibited only modest delays in development. Of these three species, only *C. septempunctata* demonstrated a lower adult weight when feeding on L‐strain aphids, which may be indicative of lower adult fitness (Honěk, 1993). The remaining two beetle species, *Hi. convergens* and *An. labiculata*, performed poorly on L‐strain *A. craccivora*, but also performed poorly on A‐strain *A. craccivora*. For these two beetle species, few larvae reached adulthood on either *A. craccivora* strain, and the survivors exhibited substantially reduced adult size when compared to beetles reared on *Ac. pisum* control aphids. Thus, *A. craccivora* in general appears to be an alternate rather than essential food source for these two beetle species (Hodek & Evans, 2012). It should be noted, however, that even on *Ac. pisum*, survival to adulthood was low for both beetle species (35‐50%). It is not unusual for *Hi. convergens* to exhibit relatively low survival on pea aphids in the laboratory (e.g., Costopoulos, Kovacs, Kamins, & Gerardo, 2014), suggesting that beetle sensitivity to laboratory‐rearing conditions may have contributed to poor survival in general and exacerbated the negative effects of *A. craccivora*.

All the beetle species in the present study were more capable of using L‐strain *A. craccivora* than the multicolored Asian ladybeetle, *H. axyridis*. In a previous study, we found that *H. axyridis* invariably failed to complete larval development on L‐strain *A. craccivora*, typically dying within a few days and without ever molting (White et al., 2017). In contrast, *H. axyridis* completed larval development and reproduced normally when fed A‐strain *A. craccivora* (White et al., 2017). The present study shows that this dramatic variation in ability to consume the aphid strains is not a widespread phenomenon among lady beetles. While L‐strain *A. craccivora* seemed to be a poorer food source than A‐strain *A. craccivora* for all tested beetle species, the fitness differential between beetles fed on L‐strain and A‐strain aphids was generally quite subtle. These results deepen the mystery regarding the mechanism of L‐strain aphid toxicity for *H. axyridis*. Previous work has eliminated aphid host plant and facultative bacterial symbionts as explanatory factors (White et al., 2017). The present study indicates that the toxic effect is particular to certain predator species and that comparative mechanistic investigations between susceptible and nonsusceptible predator species may be informative.

Regardless of mechanism, the consequence for L‐strain *A. craccivora* is that its “toxic” trait may act as a narrow‐spectrum defense, negatively affecting some but not all enemy species. How common and effective such partial defenses might be for herbivores remains to be seen. There are certainly other aphid examples in which individuals are protected from some enemies but vulnerable to others (e.g., Asplen et al., 2014; Cayetano & Vorburger, 2015; Michaud, 2000; Müller, Adriaanse, Belshaw, & Godfray, 1999). Whether an herbivore population will benefit from such narrow‐spectrum defenses ultimately depends on the composition of susceptible versus less susceptible enemies in the local community (Lenhart & White, 2017), and the degree to which compensatory attack by less susceptible enemies undercuts the defensive virtue of the selectively toxic trait for the herbivore (Letourneau, Jedlicka, Bothwell, & Moreno, 2009).

In turn, narrow‐spectrum herbivore defenses have the potential to structure the predator community. Consumers that are able to exploit such resources have access to a niche that susceptible consumers do not, and may benefit from reduced interspecific competition and reduced intraguild predation (Jeffries and Lawton 1984, Snyder, 2009). Such benefits would be particularly likely in systems where the susceptible enemy is otherwise dominant, as is the case *H. axyridis* (Hesler, Kieckhefer, & Catangui, 2004). Prey such as L‐strain *A. craccivora* represent a niche that cannot be substantially exploited by *H. axyridis*, an aggressive invasive species that typically outcompetes and consumes many other species of coccinellids (Koch, 2003; Lucas et al., 2002; Snyder, Clevenger, & Eigenbrode, 2004; Ware & Majerus, 2008). In theory, it is possible that selectively toxic prey allow for resource partitioning (Chesson, 2000) and represent a window of opportunity for subdominant predators to persist in communities where *H. axyridis* has invaded. Future empirical efforts should test how differential prey toxicity affects predator community assembly in the field and investigate whether this process may predict lady beetle community resilience to *H. axyridis* invasion.

In conclusion, our research highlights that prey can exhibit intraspecific heterogeneity in toxicity (White et al., 2017) and that generalist predator species can be heterogeneous in their sensitivity to such prey. Together, these findings suggest that niche partitioning among generalist predators may be more nuanced than previously appreciated and that dynamic ecological and evolutionary interactions between generalist predators and their prey may be ongoing.

## CONFLICT OF INTEREST

None declared.

## AUTHOR CONTRIBUTIONS

KAJ and JSM collected the data, KAJ and JAW analyzed the data and wrote the initial draft of the article, and all authors revised and approved of the final version.
